# Use of Artificial Intelligence in the Early Diagnosis of Oral Cancer: Current Update, Limitations and Future Perspectives 

**DOI:** 10.30476/dentjods.2025.105757.2609

**Published:** 2026-06-01

**Authors:** Manas Bajpai

**Affiliations:** 1 Dept. of Oral Pathology and Microbiology, Rural Dental College, Loni, Maharashtra, India.

**Keywords:** Artificial intelligence, Oral cancer, Squamous cell carcinoma, Oral health

## Abstract

Oral cancer represents a significant global health challenge with rising incidence and mortality rates in various regions. The prognosis of oral cancer significantly increases if it is diagnosed in early stages. Artificial intelligence (AI) has demonstrated significant potential in enhancing early oral cancer diagnosis through various applications, revolutionizing the field of oral pathology. These innovative approaches are transforming the way healthcare professionals detect, asses and mange the cases of oral cancer cases, potentially leading to improved patient outcomes and reduced mortality rates. This brief communication explores the integration AI in the early diagnosis of oral cancer, highlighting recent advancements and potential future applications. The study reviews current methodologies employing AI technologies, such as machine learning and deep learning algorithms, to enhance the accuracy and efficiency of oral cancer detection. It discusses the role of AI in analyzing clinical images, patient data, and other diagnostic tools, emphasizing its ability to identify precancerous lesions and reduce diagnostic delays.

## Introduction

The advent of artificial intelligence (AI) in medical diagnostics has heralded a transformative era, particularly in the early detection of oral cancer, a condition often associated with high morbidity and mortality rates [ 1 ]. Early diagnosis is paramount in improving patient outcomes, as the survival rate for oral cancer significantly increases when detected at its initial stages [ 1 - 2 ]. Traditional diagnostic methods, while valuable, often suffer from limitations such as subjective interpretations and the necessity for invasive procedures. In contrast, AI technologies, including machine learning algorithms and image recognition systems, offer innovative solutions by enhancing accuracy and efficiency in identifying potential malignancies [ 3 - 5 ]. By analyzing vast datasets and recognizing patterns indiscernible to human observers, AI not only aids in prompt diagnosis but also holds the promise of reducing healthcare costs and optimizing clinical workflows. Thus, exploring the role of AI in oral cancer diagnostics is crucial for understanding its potential to revolutionize contemporary medical practices [ 4 , 6 ].

### Overview of oral cancer and its significance in public health

Oral cancer, primarily manifesting as oral squamous cell carcinoma (OSCC), is a significant public health concern, particularly given its rising incidence and mortality rates [ 2 ]. While it comprises a relatively small share of malignant neoplasms, its impact is profound, especially in regions such as the South East Asia, where current epidemiological data on precancerous lesions remain sparse and unreliable [ 2 , 5 - 7 ]. Effective management relies heavily on early diagnosis, yet traditional screening methods, such as visual examinations, lack evidence supporting their efficacy in significantly reducing mortality or preventing disease progression [ 8 ]. This highlights an urgent need for innovative diagnostic approaches. The application of AI in this domain may offer transformative potential; AI and machine learning have demonstrated promising capabilities in improving diagnostic precision and prognostic predictions for OSCC, underscoring their significance in public health strategies. Embracing these technologies could redefine clinical practices and enhance patient outcomes [ 8 - 10 ].

### The Role of Artificial Intelligence in Medical Diagnostics

The role of AI into medical diagnostics has the potential to revolutionize early detection methods, particularly in the context of oral cancer [ 4 - 5 , 10 ]. By leveraging advanced techniques such as deep learning and neural networks, AI can significantly enhance diagnostic precision, surpassing traditional methodologies [ 10 - 12 ]. For instance, studies indicate a marked increase in AI-related publications focusing on dental applications, highlighting a trend toward improved accuracy in detecting various pathologies, including oral cancer [ 6 ]. This evolution is critical, as timely diagnosis can drastically influence patient outcomes [ 4 , 10 ]. Additionally, the accessibility of electronic clinical data and big data analytics allows for the comprehensive analysis necessary to implement AI solutions effectively within healthcare systems. Coupled with a commitment to ethical standards, AI not only fosters better clinical outcomes but also aligns with the overarching goal of elevating patient care in the dental field [ 11 ].

### Overview of AI technologies used in healthcare

**Figure 1:**
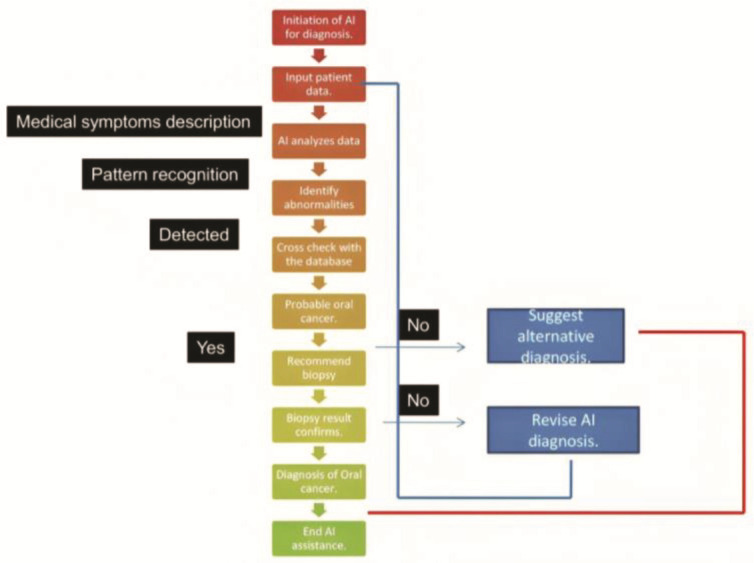
The schematic chart shows an algorithm that can be used for the diagnosis of oral cancer with AI

AI technologies have significantly transformed health care, particularly in diagnostic sectors such as oral cancer detection [ 13 ]. The integration of machine learning, particularly through algorithms like convolutional neural networks (CNNs) and artificial neural networks (ANN-s), facilitates enhanced analysis of radiographic images, enabling earlier and more accurate diagnosis of conditions, including oral cancers [ 14 - 16 ]. These advancements enhance not only the diagnostic precision but also streamline the workflow for healthcare professionals, addressing the increasing demand for efficient patient care (Figure 1). With the rapid increase in AI-related research in the last five years, applications of AI extend to personalized treatment planning, predictive analytics, and real-time decision support during clinical procedures. Consequently, AI is not merely an auxiliary tool but is becoming integral to redefining clinical practices and improving patient outcomes, solidifying its role in the early diagnosis and management of oral cancer [ 10 , 14 , 17 ]. 

### Applications of AI in Oral Cancer Detection

The integration of AI technologies in oral cancer detection represents a significant advancement in medical diagnostics, offering enhanced accuracy and efficiency. AI, particularly through machine learning algorithms, enables the analysis of radiographic and optical images to identify malignant lesions at early stages, thereby improving patient outcomes [ 14 - 15 , 18 ]. The deployment of AI in this realm not only facilitates automated diagnostic processes, reducing the burden on healthcare professionals, but also minimizes human error associated with traditional methods [ 14 ]. For example, advancements in dental imaging powered by AI have shown the potential to streamline diagnosis and treatment planning, helping to reduce costs and time considerably [ 17 - 19 ]. However, it is crucial to ensure the correct application of these technologies; improper use of genetic algorithms, for instance, can lead to suboptimal solutions in medical diagnosis, highlighting the need for rigorous implementation and training in AI methodologies [ 19 ].

### Case studies demonstrating AI's effectiveness in early diagnosis

The integration of AI in the early diagnosis of oral cancer has been significantly demonstrated through various case studies showcasing its superior diagnostic capabilities compared to traditional methods. For instance, the application of ANNs has proven effective in predicting health outcomes, such as differentiating between benign and malignant oral lesions with an impressive accuracy of 87.3% in specific studies [ 20 ]. Furthermore, recent literature highlights the remarkable advancements in AI technologies including deep learning and CNNs, which have dramatically enhanced radiographic analysis and diagnostic precision in dentistry. These innovations not only facilitate timely interventions but also optimize treatment planning, significantly improving patient prognosis [ 14 , 18 , 20 - 21 ]. Collectively, these case studies underscore the transformative potential of AI technologies in revolutionizing early detection capacities, ultimately leading to better clinical outcomes in oral cancer management [ 19 ].

### Limitations of AI in Diagnosis

The integration of AI in the early diagnosis of oral cancer holds great promise; however, numerous technical limitations hinder its efficacy and clinical applicability [ 17 ]. Despite advancements in machine learning algorithms, such as support vector machines and ANNs, the variability in specificity and sensitivity across studies remains a significant concern, indicating inconsistent diagnostic performance in different clinical settings. Additionally, the complexity of oral cancer’s heterogeneous biological characteristics poses challenges for AI systems striving for accurate classification and prediction [ 14 - 17 ]. The reliance on large datasets for training these models often leads to issues of over- fitting, which diminishes their generalizability to new cases [ 22 ]. Furthermore, the lack of standardization in data collection and analysis methods complicates the integration of AI tools into everyday clinical practice, ultimately impeding the widespread adoption of these technologies in the fight against oral cancer [ 16 , 19 , 21 - 22 ].

### Challenges in Data Quality and Quantity

The effectiveness of AI in the early diagnosis of oral cancer is significantly hindered by challenges related to data quality and quantity [ 22 - 23 ]. High-performing AI algorithms, particularly those employing machine learning techniques, rely on vast datasets that are both diverse and representative to train effectively [ 22 ]. However, many studies indicate that the available data for OSCC is often limited, potentially skewed, and lacking in comprehensive demographic representation, which can lead to inadequate model performance and misdiagnosis. Furthermore, the quality of the data utilized, including images and clinical outcomes, must be meticulously curated to ensure accuracy and reliability; any inconsistencies can severely compromise the diagnostic power of AI systems consequently, addressing these challenges is critical for enhancing the validity and clinical applicability of AI in oral cancer diagnosis [ 21 - 23 ].

### Ethical and Social Implications

The incorporation of AI in early oral cancer diagnosis presents significant ethical and social implications that warrant careful examination [ 1 - 2 , 11 , 14 , 22 ]. While AI technologies promise enhanced diagnostic accuracy, they also raise concerns regarding accountability and patient autonomy. The reliance on machine learning algorithms, particularly for critical health decisions, challenges traditional clinician-patient relationships, necessitating a discussion about who is responsible when these systems fail or produce erroneous results. Furthermore, the accessibility of such technologies can exacerbate existing health disparities, creating a societal divide where only certain populations benefit from advanced AI diagnostics while others remain underserved [ 22 ]. There is a pressing need to address these ethical dilemmas through regulatory frameworks that ensure transparency, explainability, and equitable access to AI systems in healthcare, as highlighted by recent studies that underline both the potential and limitations of machine learning in this context [ 16 ].

### Concerns Regarding Patient Privacy and Consent

In the context of AI utilization for early oral cancer diagnosis, concerns surrounding patient privacy and consent have emerged as significant barriers [ 14 - 19 ]. As healthcare systems increasingly transition from a disease-centric to a patient-centric approach, safeguarding patient data becomes crucial. The nature of AI analysis often involves processing vast amounts of sensitive information, raising fears of potential data breaches and unauthorized access [ 23 ]. Moreover, ethical considerations surrounding informed consent are paramount; patients must fully understand how their data will be used, stored, and shared. Without stringent privacy measures, trust in AI applications can erode, impeding their integration into clinical practice [ 22 - 23 ]. This situation is exacerbated by existing systemic challenges, such as complacency towards privacy issues and a lack of robust policies governing data use in healthcare, which further complicates the ethical landscape of AI in this sensitive field. Addressing these concerns is essential to ensure both the efficacy of AI tools and the protection of patient rights [ 18 , 21 ].

### Future implications of AI in improving oral cancer outcomes

The future implications AI in enhancing oral cancer outcomes are profoundly promising, especially as technology continues to evolve [ 2 - 9 ]. AIs capacity for rapid data analysis, combined with machine learning algorithms, positions it as a crucial ally in early detection and diagnosis [ 15 ]. By harnessing vast datasets, AI can identify patterns and risk factors that may elude human practitioners, resulting in more accurate preliminary assessments [ 8 , 17 ]. Furthermore, AI integration into imaging technologies promises to enhance the visualization of lesions, enabling earlier and more precise interventions [ 11 , 24 - 26 ]. As research advances, the potential for personalized treatment plans driven by AI insights could transform patient prognoses and reduce morbidity rates significantly. Lastly, the incorporation of AI in public health initiatives may lead to improved awareness and screening processes, ensuring that individuals at high risk receive timely care [ 17 , 26 - 28 ]. Collectively, these developments could mark a substantial shift in the fight against oral cancer. The conclusion derived from the investigation into the use of AI in the early diagnosis of oral cancer emphasizes its transformative potential in clinical practice [ 11 , 23 , 29 - 31 ]. By mitigating the delays associated with traditional diagnostic methods, AI can enhance the accuracy and efficiency of screenings, ultimately leading to earlier interventions and improved survival rates for patients [ 32 ]. Numerous studies affirm the technological capabilities of AI in detecting oral cancer, although challenges such as data quality, external validation, and ethical considerations remain prevalent [ 24 , 30 - 31 ]. Addressing these issues is essential for the responsible integration of AI into healthcare systems. Additionally, the interdisciplinary collaboration between dental professionals and AI specialists is crucial for maximizing the efficacy of these innovations [ 33 ]. 

## Conclusion

It can be concluded that with appropriate frameworks in place, AI could significantly advance the landscape of oral cancer diagnostics and patient care, shaping better health outcomes in this critical area.
